# Serologic Evidence for the Exposure of Eastern Coyotes (*Canis latrans*) in Pennsylvania to the Tick-Borne Pathogens Borreliella burgdorferi and Anaplasma phagocytophilum

**DOI:** 10.1128/mSphere.00544-20

**Published:** 2020-08-12

**Authors:** Jerilyn R. Izac, Andrew C. Camire, Edward J. A. Schuler, Amanda L. Hatke, Nathaniel S. O’Bier, Lee D. Oliver, Avery Corondi, Olivia C. Plocinski, Russell P. Desmond, Waheeda A. Naimi, Jason A. Carlyon, Kyle R. Van Why, Jennifer Shelly, Richard T. Marconi

**Affiliations:** a Department of Microbiology and Immunology, Virginia Commonwealth University Medical Center, Richmond, Virginia, USA; b Department of Animal Biotechnology and Conservation, Delaware Valley University, Doylestown, Pennsylvania, USA; c USDA-APHIS-Wildlife Services, Harrisburg, Pennsylvania, USA; University of Kentucky

**Keywords:** *Anaplasma*, *Borrelia*, *Borreliella*, coyote, *Ixodes*, Lyme disease, *Canis latrans*, DbpA, Eastern coyotes, P44, VlsE, canines

## Abstract

Lyme disease and anaplasmosis are tick-borne bacterial diseases caused by *Borreliella* and *Anaplasma* species, respectively. A comprehensive analysis of the exposure of eastern coyotes (Canis latrans) in the northeastern United States to tick-borne pathogens has not been conducted. In this report, we assess the serological status of 128 eastern coyotes harvested in Pennsylvania in 2015 and 2017 for antibodies to Borreliella burgdorferi and Anaplasma phagocytophilum. Immunoblot and dot blot approaches were employed to test each plasma sample by using cell lysates and recombinant proteins as detection antigens. The results demonstrate high seropositivity incidences of 64.8% and 72.7% for B. burgdorferi and A. phagocytophilum, respectively. Antibodies to both pathogens were detected in 51.5% of the plasma samples, indicating high potential for coinfection. Antibodies to the B. burgdorferi proteins DbpB, VlsE, DbpA, BBA36, and OspF (BBO39) were detected in 67.2, 63.3, 56.2, 51.6, and 48.4% of the plasma samples, respectively. Antibodies to the A. phagocytophilum P44 and P130 proteins were detected in 72.7 and 60.9% of the plasma samples, respectively.

**IMPORTANCE** The incidence of Lyme disease (Borreliella burgdorferi) and anaplasmosis (Anaplasma phagocytophilum) are increasing in North America and Europe. The causative agents of these debilitating tick-transmitted infections are maintained in nature in an enzootic cycle involving *Ixodes* ticks and diverse mammals and birds. It has been postulated that predators directly or indirectly influence the dynamics of the enzootic cycle and disease incidence. Here, we demonstrate high seropositivity of eastern coyotes for B. burgdorferi and A. phagocytophilum. As coyotes become established in urban and suburban environments, interactions with humans, companion animals, and urban/suburban wildlife will increase. Knowledge of the pathogens that these highly adaptable predators are exposed to or carry, and their potential to influence or participate in enzootic cycles, is central to efforts to reduce the risk of tick-borne diseases in humans and companion animals.

## Abstract (precis)

The incidence of Lyme disease (Borreliella burgdorferi) and anaplasmosis (Anaplasma phagocytophilum) are increasing in North America and Europe. The causative agents of these debilitating tick-transmitted infections are maintained in nature in an enzootic cycle involving *Ixodes* ticks and diverse mammals and birds. It has been postulated that predators directly or indirectly influence the dynamics of the enzootic cycle and disease incidence. Here, we demonstrate high seropositivity of eastern coyotes for B. burgdorferi and A. phagocytophilum. As coyotes become established in urban and suburban environments, interactions with humans, companion animals, and urban/suburban wildlife will increase. Knowledge of the pathogens that these highly adaptable predators are exposed to or carry, and their potential to influence or participate in enzootic cycles, is central to efforts to reduce the risk of tick-borne diseases in humans and companion animals.

## OBSERVATION

The incidence of tick-borne diseases (TBDs) is increasing throughout North America and Europe (1, 2). In the eastern half of North America, the causative agents of Lyme disease (Borreliella burgdorferi) and anaplasmosis (Anaplasma phagocytophilum) are transmitted to mammals by Ixodes scapularis ticks. Established *Ixodes* populations have been reported in >50% of U.S. counties (1) and in pockets of western and eastern Canada (3). The spread of *Ixodes* tick populations has been attributed to climate change, land use patterns, landscape, food supply (acorn abundance), predator/prey relationships, and the population of mammalian and bird reservoirs (4). Tick-borne pathogens are maintained in nature by numerous mammalian species and birds. While Peromyscus leucopus (white-footed mouse) is often cited as the primary reservoir, evidence suggests that inconspicuous hosts, including shrews, play an even greater role in the maintenance of some tick-borne pathogens in nature (5). The identification of all potential reservoirs for TBDs is central to efforts that seek to interrupt their enzootic cycles in nature and thus decrease risk of disease in humans, companion animals, and wildlife.

Predators have been postulated to influence the dynamics of the tick-mammal enzootic cycles of TBDs (6). In the northeastern United States, the population of eastern coyotes (Canis latrans) has been steadily growing due in part to the extirpation of the eastern gray wolf (Canis lupus) (7) and the ability of these wild canids to rapidly adapt to suburban and urban environments. Coyotes are aggressive apex predators that displace, attack, and kill smaller predators, including the red fox (Vulpes vulpes) (8). In areas where coyotes are thriving and red foxes are declining, the infection prevalence of *Ixodes* nymphs for B. burgdorferi is increasing (4). This is due in part to the differing predation strategies of coyotes and red foxes. Red foxes are aggressive hunters that stockpile prey for future consumption. In contrast, coyotes hunt only when hungry and do not cache their kill. Hence, as coyote populations expand and red fox populations decline, an increase in low trophic zone mammalian hosts is expected, which will in turn lead to an increased risk for TBDs (6). The goal of this study was to conduct a comprehensive assessment of the serological status of eastern coyotes for B. burgdorferi and A. phagocytophilum.

**FIG 1:**
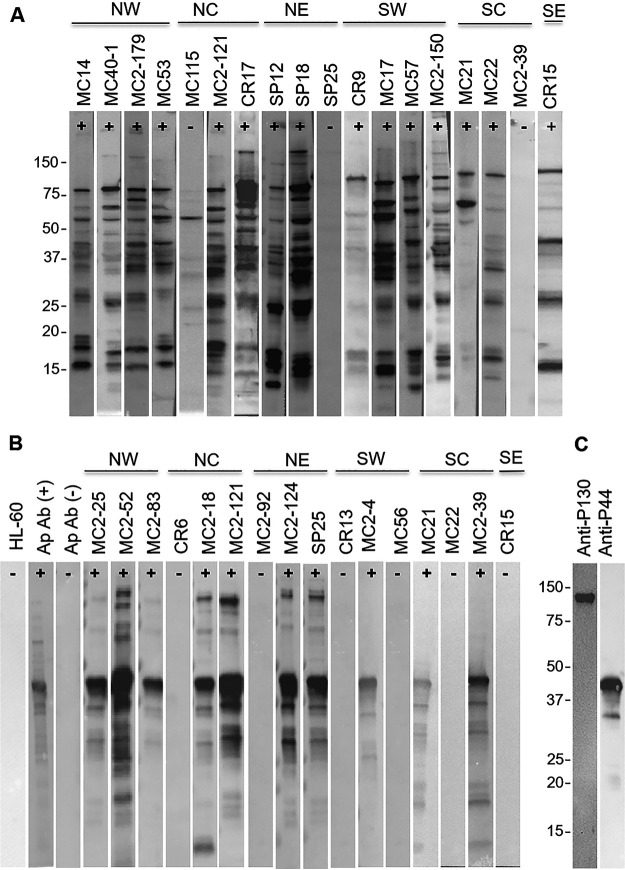
High seropositivity for B. burgdorferi and A. phagocytophilum in eastern coyotes. B. burgdorferi B31 and human promyelocytic HL-60 cells (CCL-240; ATCC) and HL-60 cells infected with A. phagocytophilum NCH-1 were cultivated as previously described (14, 15). Cells were harvested by centrifugation, washed, and solubilized in SDS-PAGE buffer. The B. burgdorferi cell lysates (A) and A. phagocytophilum-infected HL-60 cells (B) were separated by SDS-PAGE (AnykD Criterion precast gels; Bio-Rad), immunoblotted, and screened with a 1:1,000 dilution of each plasma sample, as previously described (15). The sector of Pennsylvania from which each animal was harvested is indicated above the immunoblots, with a plus or minus indicating the Ab scoring for each sample (see Table 1, footnote b, for sector abbreviations). The migration positions of native A. phagocytophilum P130 and P44 proteins were determined by screening cell lysate immunoblots with rat anti-P130 and rat anti-P44 antisera (C), generated as previously described (15). Molecular weight markers are indicated.

Plasma samples from 128 eastern coyotes were screened for antibodies (Abs) to B. burgdorferi and A. phagocytophilum by using cell lysate immunoblot and recombinant protein dot blot approaches. The plasma samples were collected from coyotes harvested in U.S. Department of Agriculture (USDA)-sanctioned hunting and trapping events in the Commonwealth of Pennsylvania during 2015 and 2017 (Special Use: Scientific Study Permit no. 48548). All animal procedures were conducted in accordance with the *Guide for the Care and Use of Laboratory Animals* and in congruence with protocols approved by the Virginia Commonwealth University (VCU) Institutional Animal Care and Use Committee. Information on the collection sites, sex, and developmental stage of each animal is provided in Table 1. The initial screen for Abs to B. burgdorferi was done by screening individual immunoblot strips of cell lysates of B. burgdorferi strain B31 with all 128 plasma samples. Seventy-five of the 128 samples (58.6%) were seropositive for several B. burgdorferi proteins (Fig. 1A, representative data). To test for Abs to A. phagocytophilum, immunoblot strips of cell lysates of HL60 cells infected with A. phagocytophilum strain NCH-1 were screened. An initial screening of 19 plasma samples revealed that 73.7 and 57.9% were Ab positive for 44- and 130-kDa proteins, respectively. Screening of cell lysate immunoblot strips and recombinant P44 and P130 with antigen-specific antisera verified the identities of these immunoreactive proteins as the well characterized P44 (9) and P130 (10) antigens (Fig. 1C). It is important to note that while the actual molecular weight of P130 is 66.1 kDa, it migrates aberrantly upon SDS-PAGE due to its acidic pI of 3.8 (10).

**TABLE 1:** Sample collection information and summary of immunoblot and dot blot data

Plasma sample ID	Sex^*a*^	County (state sector)^*b*^	Date (mo/day/yr)	Longitude	Latitude	Age status^*c*^	A. phagocytophilum Ab result for P44/P130^*d*^	B. burgdorferi Ab result by IB/DB^*e*^
MC2-121	M	Clinton (NC)	2/19/17	41.178125	−77.433313	A	+/+	+/+
MC2-174	NR	NR	2/19/17	NR	NR	NR	−/−	+/+
MC2-179	F	Warren (NW)	2/19/17	41.935572	−79.537863	A	−/−	+/+
SP0	NR	NR	NR	NR	NR	NR	+/+	+/+
SP2	M	Wyoming (NE)	1/23/15	41.514487	−75.846361	SA	+/+	+/+
SP3	M	Luzerne (NE)	1/23/15	41.178429	−76.237376	A	+/+	+/+
SP10	F	Susquehanna (NE)	1/24/15	41.669079	−75.913765	A	+/+	+/+
SP11	M	Wayne (NE)	1/24/15	41.730075	−75.388202	A	+/+	−/−
SP12	F	Wayne (NE)	1/24/15	41.730075	−75.388202	A	+/+	+/+
SP14	F	Wayne (NE)	1/24/15	41.730075	−75.388202	A	+/+	+/+
SP15	F	Wayne (NE)	1/24/15	41.730075	−75.388202	A	+/+	−/+
SP17	M	Susquehanna (NE)	1/24/15	41.678991	−76.062398	SA	+/+	+/+
SP18	M	Bradford (NE)	1/24/15	41.667297	−76.26158	A	+/+	+/+
SP20	M	Wyoming (NE)	1/25/15	41.614243	−76.046592	A	+/+	+/+
SP21	M	Luzerne (NE)	1/25/15	41.113643	−75.722647	A	+/+	+/+
SP24	F	Susquehanna (NE)	1/25/15	41.792753	−75.689743	A	−/−	−/−
SP25	M	Susquehanna (NE)	1/25/15	41.669079	−75.913765	A	+/+	+/+
SP26	F	Wyoming (NE)	1/25/15	41.485811	−75.842651	SA	+/+	+/+
SP19	F	Bradford (NE)	1/24/15	41.667297	−76.26158	A	+/+	−/+
MC12	M	Clarion (NW)	2/22/15	41.319663	−79.391605	J	+/+	+/+
MC14	M	Clarion (NW)	2/22/15	41.319663	−79.391605	SA	+/+	+/+
MC15	M	Clearfield (NC)	2/22/15	41.164035	−78.384487	A	+/+	+/+
MC16	M	Clearfield (NC)	2/22/15	40.948667	−78.478513	A	+/+	+/+
MC17	F	Washington (SW)	2/22/15	40.166354	−80.259005	J	+/+	+/+
MC18	M	Erie (NW)	2/22/15	42.051004	−79.942131	SA	−/−	−/−
MC19	M	Erie (NW)	2/22/15	41.935799	−80.224812	SA	−/−	−/−
MC21	M	Cumberland (SC)	2/22/15	40.203461	−77.309962	A	+/+	+/+
MC22	M	Cumberland (SC)	2/22/15	40.314718	−76.98066	SA	−/−	+/+
MC26	M	Clearfield (NC)	2/22/15	40.947123	−78.214029	A	+/+	+/+
MC28	F	Clearfield (NC)	2/22/15	41.026246	−78.31673	J	+/+	+/+
MC30	M	Potter (NC)	2/22/15	41.758617	−78.132091	A	+/+	−/−
MC32	F	Fayette (SW)	2/22/15	40.016783	−79.588829	J	−/−	−/−
MC35	M	Beaver (SW)	2/22/15	40.589347	−80.225357	J	+/+	−/−
MC40-1	M	Erie (NW)	2/22/15	42.000261	−80.318307	A	−/−	+/+
MC40-2	M	Centre (NC)	2/22/15	40.847732	−77.686139	SA	−/−	+/+
MC48	M	Tioga (NC)	2/22/15	41.875854	−77.401458	SA	+/+	+/+
MC49	M	Mc Kean (NC)	2/22/15	41.812173	−78.480503	A	−/−	−/+
MC53	F	Clinton (NC)	2/22/15	41.384684	−77.545175	J	+/+	+/+
MC56	M	Washington (SW)	2/22/15	40.263001	−80.187993	A	−/−	+/+
MC57	F	Allegheny (SW)	2/22/15	40.382434	−80.116141	SA	−/−	+/+
MC86	F	Northumberland (NE)	2/22/15	40.75544	−76.533517	A	+/+	+/+
MC87	F	Pike (NE)	2/22/15	41.463888	−75.155292	A	+/+	+/+
MC90	M	Wyoming (NE)	2/22/15	41.525364	−75.842013	A	+/+	+/+
MC97	F	Mercer (NW)	2/22/15	41.157502	−80.089206	A	−/−	−/−
MC99	F	Mercer (NW)	2/22/15	41.186374	−80.354815	J	−/−	+/+
MC100	M	Mercer (NW)	2/22/15	41.186374	−80.354815	A	−/−	−/−
MC113	F	Butler (NW)	2/22/15	41.157342	−79.798464	SA	+/−	−/−
MC114	M	Butler (NW)	2/22/15	41.132894	−79.852167	A	+/+	+/+
MC115	F	Tioga (NC)	2/22/15	41.748528	−77.301304	A	+/+	+/−
MC130	NR	NR	2/22/15	NR	NR	NR	−/−	−/−
MC131	NR	Erie (NW)	2/22/15	41.942181	−79.985389	A	−/−	+/+
MC148	F	Clarion (NW)	2/22/15	41.125935	−79.558499	J	+/+	−/−
MC2-1	M	Clinton (NC)	2/17/17	41.07909	−77.412819	SA	+/+	−/−
MC2-2	M	Clearfield (NC)	2/17/17	40.99839	−78.341406	A	+/+	+/+
MC2-3	F	Susquehanna (NE)	2/17/17	41.267904	−78.156443	A	+/+	+/+
MC2-4	F	Indiana (SW)	2/17/17	40.486455	−79.451436	SA	+/+	−/−
MC2-5	M	Susquehanna (NE)	2/17/17	41.724059	−75.554157	A	+/+	−/−
MC2-6	M	Centre (NC)	2/17/17	41.030891	−77.949449	J	+/+	+/+
MC2-8	F	Northumberland (NE)	2/17/17	40.961519	−76.664659	A	+/+	+/+
MC2-10	F	Crawford (NW)	2/17/17	41.63794	−80.83697	A	−/−	−/−
MC2-11	F	Crawford (NW)	2/17/17	41.63794	−80.83697	A	−/−	−/+
MC2-13	M	Crawford (NW)	2/17/17	41.751677	−80.368226	J	+/+	+/+
MC2-16	M	Crawford (NW)	2/17/17	41.63794	−80.83697	A	+/+	−/−
MC2-18	F	Elk (NC)	2/17/17	41.408709	−78.434756	A	+/+	+/+
MC2-19	M	Clearfield (NC)	2/17/17	41.198563	−78.770151	A	+/+	−/−
MC2-20	F	Clearfield (NC)	2/17/17	41.161638	−78.088013	J	+/+	+/+
MC2-21	M	Crawford (NW)	2/18/17	41.63794	−80.83697	SA	+/+	+/+
MC2-23	F	Centre (NC)	2/18/17	41.086628	−77.823244	A	+/+	+/+
MC2-24	F	Tioga (NC)	2/18/17	41.760218	−77.293542	SA	+/+	+/+
MC2-25	M	Warren (NW)	2/18/17	41.653763	−78.96286	A	+/+	+/+
MC2-26	M	Warren (NW)	2/18/17	41.653763	−78.96286	A	+/+	+/+
MC2-28	F	Clarion (NW)	2/18/17	41.320065	−79.391665	J	+/+	−/−
MC2-29	F	Venango (NW)	2/18/17	41.284381	−79.762418	SA	+/−	−/−
MC2-33	M	Centre (NC)	2/18/17	41.030891	−77.949449	J	+/+	+/+
CR-7	F	Columbia (NE)	2/8/15	41.139421	−76.477064	SA	+/+	−/−
CR-4	F	Clearfield (NC)	2/8/15	40.883834	−78.59515	J	+/+	−/−
CR-6	M	Tioga (NC)	2/8/15	41.876717	−76.97547	A	−/−	−/−
CR-9	F	Cambria (SW)	2/8/15	40.605372	−78.802618	SA	+/+	+/+
CR-10	M	Cambria (SW)	2/8/15	40.605372	−78.802618	A	+/+	−/−
CR-11	M	Jefferson (NW)	2/8/15	41.097298	−78.888108	A	+/+	+/+
CR-12	M	Somerset (SW)	2/8/15	40.007978	−79.078024	A	+/+	−/−
CR-13	F	Washington (SW)	2/8/15	40.743967	−80.255714	A	−/−	−/−
CR-14	F	Washington (SW)	2/8/15	40.243967	−80.255714	SA	−/−	−/+
CR-15	F	Schuylkill (SE)	2/8/15	40.548175	−76.384797	A	−/−	+/+
CR-16	M	Luzerne (NE)	2/8/15	41.051704	−76.221094	SA	+/+	−/−
CR-17	M	Luzerne (NE)	2/8/15	41.051704	−76.221094	SA	+/+	+/+
CR-18	M	Cambria (SW)	2/8/15	40.580221	−78.606667	SA	−/−	−/−
MC2-34	M	Centre (NC)	2/18/17	41.030891	−77.949449	SA	+/+	+/+
MC2-35	M	Clearfield (NC)	2/18/17	41.140848	−78.270395	SA	+/+	+/+
MC2-36	F	Bedford (SC)	2/19/17	40.206744	−78.522794	SA	−/−	+/+
MC2-37	M	Bedford (SC)	2/19/17	40.206744	−78.522794	J	+/+	+/+
MC2-39	M	Huntingdon (SC)	2/19/17	40.241508	−78.088013	SA	+/+	−/+
MC2-41	M	Warren (NW)	2/19/17	41.945424	−79.222558	SA	−/−	+/+
MC2-44	F	Monroe (NE)	2/19/17	40.855663	−75.456433	SA	+/+	+/+
MC2-47	M	Elk (NC)	2/19/17	41.427374	−78.56094	SA	+/+	−/−
MC2-48	NR	NR	2/19/17	NR	NR	NR	−/−	+/+
MC2-51	NR	NR	2/19/17	NR	NR	NR	+/−	+/+
MC2-52	M	Clarion (NW)	2/19/17	41.214251	−79.375163	A	+/+	−/−
MC2-62	F	Somerset (SW)	2/19/17	40.176389	−78.959377	A	−/−	−/−
MC2-65	M	Clearfield (NC)	2/19/17	41.284381	−79.762418	A	+/+	+/+
MC2-68	M	Northumberland (NE)	2/19/17	40.709529	−76.842472	A	+/−	+/+
MC2-70	F	Northumberland (NE)	2/19/17	40.709529	−76.842472	SA	+/+	−/−
MC2-73	M	Pike (NE)	2/19/17	41.186231	−75.305459	SA	+/+	+/+
MC2-76	F	Centre (NC)	2/19/17	40.944512	−77.444988	A	+/+	−/−
MC2-79	F	Crawford (NW)	2/19/17	41.764223	−80.367763	J	−/−	−/−
MC2-80	M	Armstrong (SW)	2/19/17	41.006628	−79.323992	J	+/+	+/+
MC2-82	F	Allegheny (SW)	2/19/17	40.58605	−80.029207	A	+/−	+/+
MC2-83	F	Clarion (NW)	2/19/17	41.214251	−79.375163	J	+/+	−/+
MC2-89	M	Sullivan (NE)	2/19/17	41.557347	−76.502521	A	+/+	+/+
MC2-92	F	Montour (NE)	2/19/17	40.974178	−76.627268	SA	+/−	−/+
MC2-95	M	Bradford (NE)	2/19/17	41.656464	−76.853293	SA	+/+	−/−
MC2-100	F	Monroe (NE)	2/19/17	40.972418	−75.219908	A	+/+	+/+
MC2-101	NR	NR	2/19/17	NR	NR	NR	−/−	−/−
MC2-104	NR	NR	2/19/17	NR	NR	NR	−/−	−/−
MC2-111	F	Crawford (NW)	2/19/17	41.860922	−80.134784	J	−/−	+/+
MC2-113	M	Crawford (NW)	2/19/17	41.860922	−80.134784	A	−/−	−/−
MC2-119	F	Erie (NW)	2/19/17	42.000334	−80.318119	A	+/−	+/+
MC2-124	M	Luzerne (NE)	2/19/17	41.319399	−76.012652	SA	+/+	+/+
MC2-125	F	Luzerne (NE)	2/19/17	41.314399	−76.012652	SA	+/+	+/+
MC2-131	F	Susquehanna (NE)	2/19/17	41.685298	−75.1562604	SA	+/+	−/−
MC2-134	F	Westmoreland (SW)	2/19/17	40.164543	−79.807261	J	−/−	−/−
MC2-141	M	Potter (NC)	2/19/17	41.872012	−77.837218	A	+/−	−/−
MC2-146	M	Crawford (NW)	2/19/17	41.63794	−80.83697	A	−/−	−/−
MC2-147	F	Crawford (NW)	2/19/17	41.718945	−80.147558	A	+/+	−/−
MC2-150	F	Cambria (SW)	2/19/17	40.618857	−78.736284	A	+/+	+/+
MC2-155	M	Centre (NC)	2/19/17	40.847563	−77.686109	A	+/+	−/−
MC2-157	NR	NR	2/19/17	NR	NR	NR	+/+	−/−
MC2-170	F	Erie (NW)	2/19/17	42.161082	−79.989011	A	+/+	+/+

a Abbreviations: M, male; F, female; NR, not reported.

b The geographic sectors of the Commonwealth of Pennsylvania from which coyotes were harvested are indicated as follows: NW, Northwest; NC, North Central; NE, Northeast; SW, Southwest; SC, South Central; SE, Southeast.

c Developmental stage (juvenile [J], subadult [SA], or adult [A]) was determined by assessment of tooth wear by wildlife biologists from the USDA and the PGC. This study was conducted under a Special Use: Scientific Study Permit (no. 48548) issued by the Pennsylvania Game Commission. NR, not reported.

d For A. phagocytophilum, the Ab screening results for the P44 and P130 proteins are indicated as positive (+) or negative (−).

e For the Ab screening for B. burgdorferi, a plus or minus indicates if an animal was positive or negative, respectively, by immunoblot (IB) and dot blot (DB) approaches.

**FIG 2:**
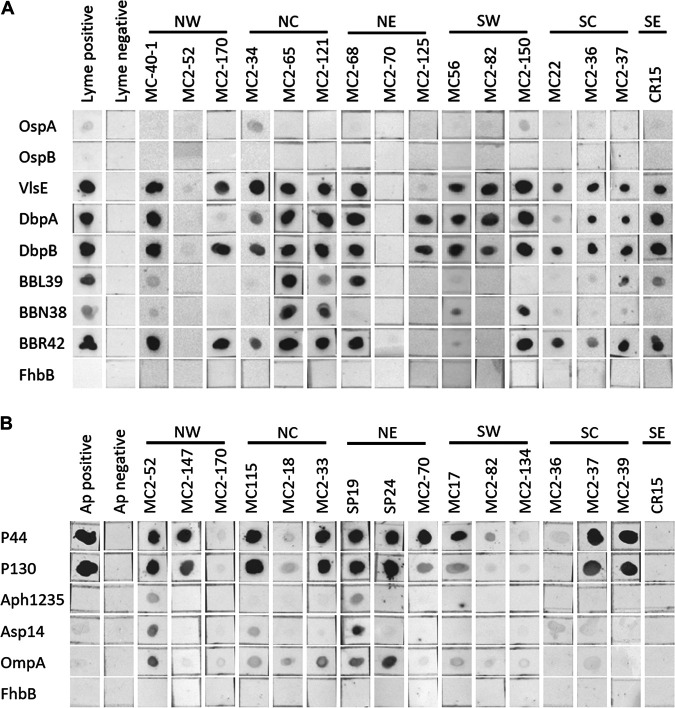
Detection of Abs to defined B. burgdorferi and A. phagocytophilum antigens. Recombinant proteins (indicated on the left) were generated by PCR amplification of B. burgdorferi B31 and A. phagocytophilum NCH-1 genomic DNA or through gene synthesis (codon optimized; GenScript). The A. phagocytophilum strain Dog2 P44 gene sequence (GenBank accession no. AGR82240.1) was used to generate recombinant P44. All primer sequences, the P44 amino acid sequence, and the *p44* codon-optimized gene sequence are provided in Table S1 in the supplemental material. The proteins were expressed from pET-45b(+) (Novagen). All cloning and protein production procedures were done as previously described (15). Dot blots were generated by spotting 125 ng of purified protein onto nitrocellulose. The membranes were air dried overnight and then blocked and screened with each plasma sample as detailed in the legend to Fig. 1. All dot blots were imaged simultaneously.

With the initial finding that a high percentage of coyotes were seropositive for B. burgdorferi and A. phagocytophilum, the entire plasma panel was screened for Abs to individual proteins that are upregulated during spirochete residence in mammals or in ticks (reviewed in reference 11). The B. burgdorferi mammalian or infection-stage proteins VlsE, DbpA, DbpB, OspE (paralogs BBL39 and BBN38), and OspF (paralog BBR42) and the tick-stage OspA and OspB proteins were produced with hexahistidine tags, purified, and screened using a dot blot format. Sixty-four percent of the plasma samples harbored Ab to at least three of the six infection-stage antigens, and 50% had Abs to all six proteins (Fig. 2; Table 2). Abs to VlsE and DbpB were detected with the highest frequency. Abs to OspA, but not OspB, were detected in 6 of the 128 samples, but the reactivity was weak and considered equivocal (Fig. 2, sample MC2-134) . This is consistent with the downregulation of OspA and OspB at the tick-mammalian interface prior to transmission to mammals (12). The FhbB protein, a factor H-plasminogen binding protein produced by the human periopathogen Treponema denticola (13), served as a negative control, and as expected, the plasma samples were not reactive with this protein. The immunoscreening results obtained with each individual plasma sample are summarized in Table 1, and the results for each specific test antigen are summarized in Table 2. In Table 3, the data are presented in terms of age, gender, collection year, and state sector.

**TABLE 2:** Recombinant proteins used as screening antigens and summary of results

Species and/or protein (B31 ORF)^*a*^	Description	% Ab-positive samples (no. of samples positive/total no.)	Reference
*B. burgdorferi*			
OspA (BBA15)	Surface lipoprotein essential for survival in ticks; produced in culture and in ticks but not in mammals	6.3 (8/128) (equivocal)	16
OspB (BBA16)	Same as described above for OspA; forms an operon with *ospA*	0 (0/128)	16
VlsE (BBF0041)	Surface lipoprotein; functions in immune evasion; not produced during cultivation or in ticks; expressed in infected mammals	63.3 (81/128)	17
DbpA (BBA24)	Decorin binding protein; facilitates dissemination during early stage infection; produced *in vitro* and *in vivo*	56.2 (72/128)	18
DbpB (BBA25)	See information for DbpA above	68.0 (87/128)	18
OspE (BBL39)	Factor H binding protein; facilitates complement evasion; all LD spirochete strains encode multiple OspE paralogs	15.6 (20/128)	19
OspE (BBN38)	See information for BBL39 above	16.4 (21/128)	20
OspF (BBR42)	Expression upregulated in mammals; Ab to OspF has been suggested to be a marker for chronic infection; most strains produce multiple OspF paralogs	43.0 (55/128)	21
OspF (BBM38)	See information for BBR42 above	40.6 (52/128)	21
OspF (BBO39)	See information for BBR42 above	48.4 (62/128)	21
Mlp (BBA36)	Surface lipoprotein; expression upregulated in mammals; Mlp protein family member	51.6 (66/128)	22
Uncharacterized protein (BBK53)	Surface lipoprotein; uncharacterized	18.8 (24/128)	
P35 (BBA73)	Function unknown; surface protein; member of paralogous protein family 54; upregulated in mammals	48.4 (62/128)	23
Protein of unknown function (BB0238)	Function unknown; required for infection in mammals	27.3 (35/128)	24
			
*A. phagocytophilum*			
P130	Unique to *A. phagocytophilum*; localizes to the vacuolar membrane	60.9 (78/128)	10
P44	Porin protein involved in immune evasion; homologs are found in *A. platys* and *A. marginale*	72.7 (93/128)	9
Aph_1235	Specific to the infectious dense core cell	3.3 (3/90)	25
Asp14	Adhesin; homologs are found in some *Anaplasma* and *Ehrlichia* species	7.8 (7/90)	26
OmpA	Adhesin; homologs are found in some *Anaplasma* and *Ehrlichia* species	18.9 (17/90)	26
			
FhbB	*T. denticola* FH binding protein; negative control	0 (0/128)	13

a The ORF designations listed are those assigned to B. burgdorferi strain B31.

**TABLE 3:** Summary of Ab screening results

Parameter	% Ab positive (no. of positive samples/total no.) for:	
	B. burgdorferi	A. phagocytophilum
Gender		
Male	65.6 (42/64)	79.7 (51/64)
Female	65.4 (36/55)	70.9 (39/55)
Not reported	55.6 (5/9)	33.3 (3/9)
Developmental stage		
Adult	71.8 (46/64)	75.0 (48/64)
Subadult	69.4 (25/36)	75.0 (27/36)
Juvenile	80.0 (16/20)	75.0 (15/20)
Not reported	62.5 (5/8)	37.5 (3/8)
State sector		
Northwest	63.6 (21/33)	57.6 (19/33)
North Central	75.9 (22/29)	86.2 (25/29)
Northeast	82.9 (29/35)	97.1 (34/35)
Southwest	58.8 (10/17)	52.9 (9/17)
South Central	100 (5/5)	60.0 (3/5)
Southeast	1/1*a*	0/1*a*
Not reported	62.5 (5/8)	37.5 (3/8)
Collection yr		
2015	68.8 (42/61)	68.9 (42/61)
2017	62.1 (41/66)	76.1 (51/66)
Total for 2015 and 2017	64.8 (83/128)	72.7 (93/128)
Total for B. burgdorferi and A. phagocytophilum	51.5 (72/128)	

^a^Due to sample size limitations, percentages would not be valid and thus were not calculated.

10.1128/mSphere.00544-20.1 TABLE S1 Oligonucleotide primer sequences, P44 protein sequence, and codon-optimized *p44* gene sequence. Download Table S1, PDF file, 0.1 MB. Copyright © 2020 Izac et al. 2020 Izac et al. This content is distributed under the terms of the Creative Commons Attribution 4.0 International license.

To screen for Abs to well characterized A. phagocytophilum proteins, recombinant P44, P130, Asp14, Aph_1235, and OmpA were generated and screened by dot blotting. Consistent with the results of the cell lysate immunoblot assays and with earlier reports that P44 is an immunodominant antigen (9), 72.7% of the plasma samples were P44 Ab positive. Ab to P130 and OmpA was detected in 60.9 and 18.9% of the plasma samples, respectively. Abs to the other proteins tested were detected in a low percentage of plasma samples (Table 2). It is important to note that Anaplasma platys, which also infects dogs and is transmitted by Rhipicephalus sanguineus ticks, produces homologs of P44. While there is significant sequence divergence between the A. phagocytophilum and A. platys P44 proteins, we cannot rule out the possibility that some animals had been infected or exposed to A. platys. However, in contrast to P44, P130 is unique to A. phagocytophilum, and thus Ab to P130 is a clear indicator of exposure to A. phagocytophilum. Note that clinical samples that may have allowed for a direct assessment of whether the individual animals were actively infected at the time of harvest were not available for analysis. However, the strong immunoreactivity of a majority of plasma samples with the cell lysates or recombinant proteins is consistent with either an active or recent infection in the animals at the time of harvest.

In summary, this study demonstrates that eastern coyotes have significant exposure to the causative agents of Lyme disease and anaplasmosis. The lifestyle habits of eastern coyotes would most certainly allow for frequent exposure to all developmental stages of *Ixodes* ticks, including larvae. This raises important questions as to the potential for coyotes to serve as reservoirs for tick-borne pathogens. While it remains to be determined if coyotes and other predators are competent reservoirs, if that proves to be the case, the results presented here have implications for the potential limitations of bait vaccine development efforts that are focused largely on targeting mice. As coyotes become increasingly urbanized and interact with humans, domestic canids, and suburban/urban wildlife, knowledge about the pathogens they may carry is important for understanding their potential contribution to the enzootic cycle of tick-borne pathogens.
