# Supplementary material for: Serologic Evidence for the Exposure of Eastern Coyotes (Canis latrans) in Pennsylvania to the Tick-Borne Pathogens Borreliella burgdorferi and Anaplasma phagocytophilum
Source: mSphere. 2020 Aug 12;5(4):e00544-20. doi: 10.1128/mSphere.00544-20 (PMC7426170; doi:10.1128/mSphere.00544-20)
Supplement: TABLE S1 [file mSphere.00544-20-st001.pdf]

Table S1. Oligonucleotide primers and P44 protein and codon optimized gene sequence.

**Protein/ORF**[a]	**Primer sequence (5'-3') and P44 protein sequence**[b]
Bb *ospA* (*bba15*) F	**GACGACGACAAGATT**AAGCAAAATGTTAGCAGCCTTGACGAGAAAAACAGC
Bb *ospA* (*bba15*) R	**GAGGAGAAGCCCGGTTTA**TTTTAAAGCGTTTTTAATTTCATCAAGTTTTG
Bb *ospB* (*bba16*) F	**ATAGTCGGATCCC**GGTGCTGAGTCAATTGGTTCC
Bb *ospB* (*bba16*) R	**ATAGTCCGGCCGTTA**TTTTAAAGCGTTTTTAAGCTCTGAAAGATTTTTAATTTC
Bb *vlsE* (*bbf0041*) F	**GACGACGACAAGATT**GCTGATAAGGACGACCCAAC
Bb *vlsE* (*bbf0041*) R	**AGGAGAAGCCCGGTT**CACTTATTCAAGGCAGGAGG
Bb *dbpA* (*bba24*) F	**ATAGTCGGATCCC**AAAATTAGATTAGAACGAAGCGCTAAAGACATTACAGATG
Bb *dbpA* (*bba24*) R	**ATAGTCCGGCCGTTA**TTAGTTATTTTTGCATTTTTCATCAGTAAAAGTAGAATTTTCC
Bb *dbpB* (*bba25*) F	**ATAGTCGGATCCC**CTTGAATCGTCCTCTAAGGATTTAAAAAACAAAATTTTAAAAATA
Bb *dbpB* (*bba25*) R	**ATAGTCCGGCCGTTA**TTATTTCTTTTTTTTGCTTTTATTATTTTTTTTCTCTCCACC
Bb *ospE* (*bbl39*) F	**GACGACGACAAGATG**CTTATAGGTGCTTGCAAG
Bb *ospE* (*bbl39*) R	**GAGGAGAAGCCCGGTTTA**TTTTAAATTTCTTTTAAGCTC
Bb *ospE* (*bbn38*) F	**GACGACGACAAGATG**CTTATAGGTGCTTGCAAAATTC
Bb *ospE* (*bbn38*) R	**GAGGAGAAGCCCGGTTTA**TTTTAAATTTTTTTTAAGCAC
Bb *ospF* (bbr42) F	**ATAGTCGGATCCC**AAAAATGATGTGACCAGCAAAGATCTGG
Bb *ospF* (bbr42) R	**ATAGTCCGGCCGTTA**CTCTTTTTTGCCTTCCACGGTCTC
Bb *ospF* (*bbm*38) F	**ATAGTCGGATCCC**AAAAACTATGCCAGCGGCGAGG
Bb *ospF* (*bbm*38) R	**ATAGTCCGGCCGTTA**TTCTTTTTTATTGCTATCTTTACTTTCTTTTTCCAGTTTC
Bb *ospF* (*bbo39*) F	**ATAGTCGGATTCC**GAACAGAACCTGGAGAGCAGCGAG
Bb *ospF* (*bbo39*) R	**ATAGTCCGGCCGTTA**TTCTTTTTTGTCTTCTTCGATGCCCTTCAG
Bb *mlp* (*bba36*) F	**ATAGTCGGATCCC**GTTAAATCGCTTACAGAAATTGATTCTGGGAATGG
Bb *mlp* (*bba36*) R	**ATAGTCCGGCCGTTA**TTAAACATTTCCATAATTTTTCAAATTTTTCAATTCGTTGTTAC
Bb (*bbk53*) F	**ATAGTCGGATCCC**CAAACTTTTTTTGAGAATTCGGAAAGTAGTGATATGGG
Bb (*bbk53*) R	**ATAGTCCGGCCGTTA**TTATGTAGGTAAAATAGAAACTGGGCT ATT ATA ATCATACCT
Bb *p35* (*bba73*) F	**ATAGTCGGATCCC**AACACAGAAGCGATAAGTGAATTACAATCAAG
Bb *p35* (*bba73*) R	**ATAGTCCGGCCGTTA**TTAGTAGTGTATGTGGTCACAACAGGTTTTTAG
Bb (*bb0238*) F	**ATAGTCGGATCCC**GATAAGCAAAAAGAGCTTGCTATTTTTTATTATGAGG
Bb (*bb0238*) R	**ATAGTCCGGCCGTTA**TCAATTTATGGAAGACAAAAACCAATTATTATCATCC
Aph *p130* F	**ATAGTCGGATCCC**TTTGAACACAATATTCCTGATACATACACAGG
Aph *p130* R	**ATAGTCCGGCCGTCA**CAACGCGAGCACGTCATC
Aph *p44* F	**GGTGATGATGATGACAAG**ATGCGTAGTCGCAGTAAACTGTTC
Aph *p44* R	**GAGGAGAAGCCCGGTTTA**ATAGCGACGGCTGCTAACGG
Aph *aph_1235* F	**ATAGTCGGATCCC**ATGAAAGGAAAGTCAGATTCTGAAATACGTACG
Aph *aph_1235* R	**ATAGTCCGGCCGCTA**ACCTTGGGTCGATGCCACAATTTTC
Aph *asp14* F	**GACGACGACAAGATT**AAGATATACCATTAGCTCCTTGGAAGAGC
Aph *asp14* R	**GAGGAGAAGCCCGGTTTA**GCTTTCTTTAGGAGTATTGGCACCGTAA
Aph *ompA* F	**GACGACGACAAGATC**TGTGGGACTCTTCTTCCAGATAGTAACG
Aph *ompA* R	**GAGGAGAAGCCCGGTTA**GTTAGCGATTGCGCTAGAGAATTC
T. denticola fhbB F	**GACGACGACAAGATT**ACTTTCAAAATGAATACTGCAC
T. denticola fhbB R	**GAGGAGAAGCCCGGTTTA**CTTTATCTTTTTGGGTAT
Aph Dog2 isolate P44 protein sequence (AGR82240.1)	MRSRSKLFLGSVMMSMAIVMAGNDVRAHDDVSALETGGAGYFYVGLDYSPAFSKIRDFSIR<br>ESNGETKAVYPYLKDGKSVKLESHKFDWNTPDPRIGFKDNMLVAMEGSVGYGIGGARVELE<br>IGYERFKTKGIRDSGSKEDEADTVYLLAKELAYDVVTGQTDNLAAALAKTSGKDIVQFANA<br>VKISSPEIDGKVCNGDHAAIKATKGKAYVAELTSTYSNEETTQCSGLGNTSQATGPKSLSG<br>FVNTVKVGEGKNWPRGRASDGSSKNIEGDPNSNAKAVATDLTKLTSDEKTIVAGLLTKTIE<br>GGEVVEIRAVSSTSVMVNACYDLLSEGLGVVPYACVGLGGNFVGVVDGHITPKLAYRLKAG<br>LSYQLSPEISAFAGGFYHQVVAAVSSRRY
Aph Dog2 isolate P44 codon optimized gene sequence	ATGCGTAGTCGCAGTAAACTGTTCCTGGGCAGCGTGATGATGAGCATGGCCATTGTGATGG<br>CAGGTAACGATGTGCGCGCACATGATGATGTGAGCGCCCTGGAAACCGGTGGTGCCGGCTA<br>TTTTTATGTGGGCCTGGACTACAGCCCGGCCTTCAGTAAGATCCGCGACTTTAGCATCCGC<br>GAAAGTAACGGCGAAACCAAAGCCGTGTACCCTTATCTGAAGGACGGCAAGAGCGTGAAGC

	TGGAGAGCCATAAATTTGACTGGAACACCCCGGATCCGCGTATCGGCTTCAAAGACAACAT<br>GCTGGTGGCCATGGAAGGTAGCGTGGGCTATGGCATTGGCGGTGCCCGTGTGGAACTGGAA<br>ATCGGCTATGAGCGCTTCAAGACCAAAGGCATTCGCGATAGTGGCAGCAAAGAGGACGAGG<br>CCGATACAGTTTACCTGCTGGCCAAAGAACTGGCCTACGATGTGGTTACCGGCCAGACCGA<br>TAATTTAGCAGCCGCCCTGGCCAAGACCAGTGGCAAAGACATCGTTCAGTTCGCCAACGCC<br>GTGAAGATTAGCAGCCCGGAGATTGATGGCAAAGTGTGCAACGGTGATCACGCCGCCATCA<br>AAGCAACCAAAGGCAAGGCATACGTGGCAGAGCTGACCAGCACCTACAGCAATGAAGAGAC<br>CACCCAGTGCAGCGGTCTGGGCAATACCAGCCAAGCAACCGGTCCGAAAAGCCTGAGCGGT<br>TTTGTGAACACAGTGAAGGTGGGCGAAGGCAAAAACTGGCCGCGCGGTCGTGCAAGTGATG<br>GCAGCAGCAAAAACATCGAGGGCGATCCGAATAGCAACGCCAAAGCAGTGGCCACCGATCT<br>GACCAAACTGACCAGCGACGAGAAAACCATCGTGGCCGGTTTACTGACCAAAACCATCGAA<br>GGCGGCGAAGTTGTGGAGATTCGCGCAGTGAGTAGTACCAGCGTGATGGTTAATGCCTGTT<br>ATGACCTGCTGAGCGAAGGCCTGGGTGTGGTTCCGTATGCCTGTGTGGGCTTAGGCGGTAA<br>CTTCGTGGGTGTGGTGGACGGTCATATTACCCCTAAGCTGGCCTATCGCCTGAAGGCCGGT<br>CTGAGCTATCAGCTGAGTCCTGAGATCAGCGCATTTGCCGGCGGCTTCTATCATCAGGTGG<br>TTGCCGCCGTTAGCAGCCGTCGCTAT

[a] Abbreviations: Bb-*B. burgdorferi*, Aph-*A. phagocytophilum*. In the first column, the ORF designations listed are those assigned to *B. burgdorferi* strain B31.

[b] bolded region included for cloning purposes
